# The association of depression and patient and resuscitation characteristics with survival after out-of-hospital cardiac arrest: a cohort study

**DOI:** 10.1093/europace/euae209

**Published:** 2024-08-06

**Authors:** Raied Alotaibi, Nynke Halbesma, Caroline A Jackson, Gareth Clegg, Remy Stieglis, Hans van Schuppen, Hanno L Tan

**Affiliations:** Usher Institute, University of Edinburgh, Edinburgh, UK; Prince Sultan College for Emergency Medical Services, King Saud University, Riyadh, Saudi Arabia; Usher Institute, University of Edinburgh, Edinburgh, UK; Usher Institute, University of Edinburgh, Edinburgh, UK; Resuscitation Research Group, The University of Edinburgh, Edinburgh, UK; Department of Anaesthesiology, Amsterdam UMC, Amsterdam, The Netherlands; Department of Anaesthesiology, Amsterdam UMC, Amsterdam, The Netherlands; Department of Clinical and Experimental Cardiology, Amsterdam UMC, University of Amsterdam, Meibergdreef 9, Amsterdam 1105 AZ, The Netherlands; Netherlands Heart Institute, Moreelsepark 1, Utrecht 3511 EP, The Netherlands

**Keywords:** Out-of-hospital cardiac arrest, Depression, Cardiopulmonary resuscitation, Mental health, Survival, Cardiovascular disease

## Abstract

**Aims:**

Out-of-hospital cardiac arrest (OHCA) is a leading cause of death worldwide, with cardiovascular disease (CVD) being a key risk factor. This study aims to investigate disparities in patient/OHCA characteristics and survival after OHCA among patients with vs. without depression.

**Methods and results:**

This is a retrospective cohort study using data from the AmsteRdam REsuscitation Studies (ARREST) registry from 2008 to 2018. History of comorbidities, including depression, was obtained from the patient’s general practitioner. Out-of-hospital cardiac arrest survival was defined as survival at 30 days post-OHCA or hospital discharge. Logistic regression models were used to obtain crude and adjusted odds ratios (ORs) for the association between depression and OHCA survival and possible effect modification by age, sex, and comorbidities. The potential mediating effects of initial heart rhythm and provision of bystander cardiopulmonary resuscitation were explored. Among 5594 OHCA cases, 582 individuals had pre-existing depression. Patients with depression had less favourable patient and OHCA characteristics and lower odds of survival after adjustment for age, sex, and comorbidities [OR 0.65, 95% confidence interval (CI) 0.51–0.82], with similar findings by sex and age groups. The association remained significant among the Utstein comparator group (OR 0.63, 95% CI 0.45–0.89) and patients with return of spontaneous circulation (OR 0.60, 95% CI 0.42–0.85). Initial rhythm and bystander cardiopulmonary resuscitation partially mediated the observed association (by 27 and 7%, respectively).

**Conclusion:**

Out-of-hospital cardiac arrest patients with depression presented more frequently with unfavourable patient and OHCA characteristics and had reduced chances of survival. Further investigation into potential pathways is warranted.

## Graphical Abstract

**Graphical Abstract:**
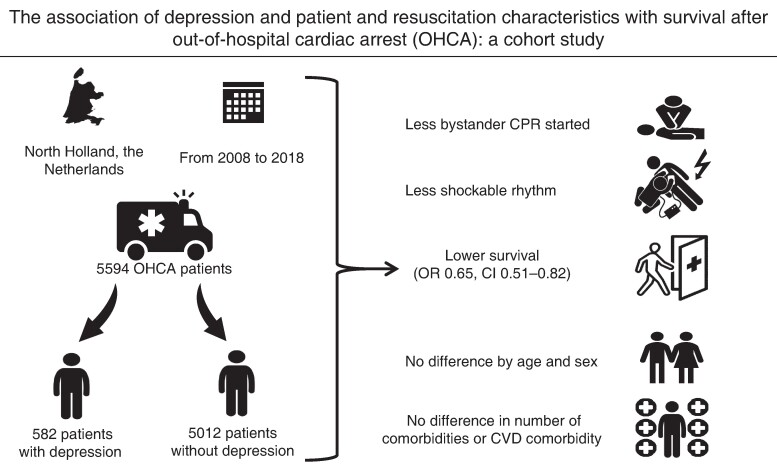
Graphical summary of the methods and main findings of the present study on the association of depression and patient and resuscitation characteristics with survival after out-of-hospital cardiac arrest (OHCA): a cohort study. CPR, cardiopulmonary resuscitation; CVD, cardiovascular disease; OR, odds ratio

**What’s new?**

- Depression is associated with lower odds of out-of-hospital cardiac arrest (OHCA) survival, even after adjusting for age, sex, and comorbidities.
- This association may be partially influenced by a lower occurrence of initial shockable rhythms and bystander cardiopulmonary resuscitation in individuals with depression.
- The association persists in witnessed OHCAs with an initial shockable rhythm and patients with return of spontaneous circulation.
- Further investigation of potential pathways between depression and lower OHCA survival is warranted.

## Background and rationale

Out-of-hospital cardiac arrest (OHCA) is one of the leading causes of death around the world.^1^ The estimated annual incidence of OHCA ranges from 30 to 97/100 000 population in Europe and other high-income countries, including the USA, Japan, and Australia.^1,2^ Survival rates after OHCA remain relatively low. For instance, the reported average rate of survival to 30 days or hospital discharge was 8% across European countries in 2017.^3^ Yet, survival rates have risen significantly over the past decade mostly in high-income countries (20–25% in some national and local registries^1,4^), largely due to the development and implementation of new resuscitation guidelines and strategies, in particular provision of cardiopulmonary resuscitation (CPR) by bystanders, telephone-assisted CPR, and increased use of automated external defibrillators (AEDs), especially in high-income countries.^5–8^ However, these strategies may not have increased survival rates in all OHCA patients.^9^ For example, one study that included 27 523 non-traumatic OHCA cases in Denmark between 2001 and 2015 reported that 30-day survival rate increased over time in patients without psychiatric illnesses but not in patients with psychiatric illnesses.^10^

Most OHCAs have an underlying cardiac aetiology, and hence, risk factors for cardiovascular diseases (CVDs), such as depression, may influence the risk of suffering OHCA and/or the chances of surviving an OHCA event.^3,11,12^ Depression and CVD are both highly prevalent conditions in the general population and have a complex bidirectional relationship.^13–16^ Over the last three decades, depression has become more prevalent, affecting over 285 million people worldwide in 2019.^17^ The occurrence of depression following cardiac arrest or other cardiovascular emergencies such as myocardial infarction affects patients’ quality of life and long-term outcomes.^18,19^ Moreover, premature mortality in people with depression is well established, with CVD the main contributory factor for excess mortality.^20^

However, little is known about whether depression is a risk factor for reduced survival rate following OHCA. To our knowledge, just one study has previously investigated this, reporting that people with depression may have lower survival rate following OHCA than people without any psychiatric illness.^10^ Replication of these findings in other settings and study populations is critical to establishing a strong evidence base on the possible link between depression and OHCA survival. In the present study, we therefore sought to compare OHCA survival rates between patients with depression and patients without depression. In addition, we examined how the distribution of factors that influence OHCA survival in the general population, such as patient characteristics (age, sex, and comorbidities)^21–23^ and OHCA characteristics (e.g. location, witness status, initial heart rhythm, bystander CPR, and collectively called Utstein factors),^11,24^ vary by depression status.

## Methods

### Study setting and population

We conducted a retrospective cohort study using data from the AmsteRdam REsuscitation Studies (ARREST) registry, including cases from the years 2008–2018. Information regarding the rationale and methodology of case identification in the ARREST registry has been described previously.^25^ Briefly, the ARREST registry is a community-based OHCA registry that includes data on all emergency medical service (EMS)–attended OHCAs from 2005 onwards in the province of North Holland, the Netherlands. The region covers 2404 km^2^ and has a population of 2.8 million people.

We excluded patients younger than 18 years, EMS-witnessed arrests, non-medical aetiology, and missing medical history information. We excluded patients with EMS-witnessed OHCA because survival chances in these patients are not influenced by provision or non-provision of community early recognition and community-based interventions, such as bystander CPR and defibrillation, which are key factors in determining survival from OHCA.^26^

Of surviving patients, a signed informed consent was obtained. Cases in which the patient declined consent or when informed consent could not be retrieved were excluded.

### Data collection

Pre-hospital data are collected prospectively for each OHCA following the Utstein guidelines.^27^ Cases are identified through data from the dispatch centre. In addition, ambulance personnel are asked to call the study centre to provide information after each attended OHCA (e.g. age, sex, location, witness status, and bystander CPR) and to send the continuous electrocardiogram data.^25^ In the Netherlands, it is mandatory to consult the general practitioner (GP) for non–life-threatening medical issues prior to seeking care from a specialist. Whenever a patient visits a medical specialist or is admitted to hospital, a detailed report of their treatment is sent to the GP, who keeps a record of the patient’s medical history. General practitioners are asked to complete a questionnaire developed by the ARREST team, which includes predefined questions about the patient’s pre-existing comorbidities.

### Exposure, outcome. and covariates

In this study, depression is defined as a dichotomous variable based on medical history information provided by GPs. The primary outcome was 30-day survival or survival to hospital discharge. Data on survival were collected by the ARREST team from the hospital of admission and verified through other data sources, including the patient’s GP.^25^

Covariates included patient characteristics such as age, sex, and pre-existing physical comorbidities. Comorbidity definition was based on the questionnaire completed by GPs and consisted of myocardial infarction, atrial arrhythmia, cardiomyopathy, congenital heart disease, ventricular arrhythmia, valvular heart disease, heart failure, angina pectoris, any cancer, rheumatic disease, renal dysfunction, liver dysfunction, diabetes mellitus type 1, and diabetes mellitus type 2. Out-of-hospital cardiac arrest event characteristics included collapse witness status, provision of bystander CPR, location of arrest, initial heart rhythm, and a return of spontaneous circulation (ROSC). We dichotomized initial heart rhythm into shockable or non-shockable rhythm, with shockable rhythm defined as ventricular fibrillation or ventricular tachycardia.

### Statistical analysis

We summarized baseline characteristics for OHCA cases with or without depression as frequencies with proportions for categorical variables and medians with interquartile range (IQR) for continuous variables and evaluated differences in baseline characteristics between groups using *χ*^2^ tests or *t*-tests. We used logistic regression models to obtain both crude (unadjusted) and adjusted odds ratios (ORs) and corresponding 95% confidence intervals (CIs) for the association between depression and OHCA survival.

We used a directed acyclic graph,^28^ incorporating insights from existing literature and subject matter expertise, to identify potential confounding variables for inclusion in the multivariable models (see Supplementary material online, *Figure S1*). These graphs also facilitated the identification of potential effect modifications and mediating variables that may lie on the causal pathway.^28,29^ A list of models showing ORs for the sequential adjustments were reported as follows: age (Model 1); age and sex (Model 2); age, sex, and number of comorbidities (Model 3); age, sex, and CVD comorbidity (Model 4), with age included as a continuous variable in all models. Furthermore, we adjusted for characteristics that may lie on the causal pathway in additional exploratory models as follows: age, sex, number of comorbidities, and CVD (Model 5); age, sex, bystander CPR, and initial rhythm (Model 6); age, sex, location of arrest, and witness status (Model 7; see Supplementary material online, *Figure S2*).

We investigated for effect modification by sex and age on the additive and multiplicative scales.^30^ We divided age into two categories: older adults (above 65 years) and younger adults (65 years and below). We followed the approach proposed by Knol and VanderWeele^30^ for assessing and presenting effect modification analysis: (i) ORs were calculated using logistic regression for each stratum of potential effect modifiers, using a single reference category; (ii) stratified ORs were presented for the association between depression and OHCA survival across categories of the potential effect modifiers; (iii) measures of effect modification on both the additive scale, using the relative excess risk due to interaction (RERI), and the multiplicative scale, using the ORs, were examined.^30^

Moreover, to investigate whether initial rhythm and bystander CPR mediate the relationship between depression and OHCA survival, we conducted a mediation analysis using the difference method.^31^ This analysis allowed the estimation of the proportions mediated, which quantifies the extent to which the total effects of depression on OHCA survival operate through a decreased probability of receiving CPR or a reduced chance of having an initial shockable rhythm. We also performed two *post hoc* sensitivity analyses: one in which we used the Utstein comparator group (OHCA cases witnessed by a bystander and having an initial shockable rhythm)^27^ and one in which we only included patients who had a ROSC. We performed this additional analysis to investigate potential differences in the association between depression and survival in these two subgroups of patients with specific OHCA characteristics known to be associated with improved survival. Furthermore, to identify possible differences in the demographic or OHCA characteristics between patients with or without GP data, we provided these data, along with outcome data, in an additional descriptive table (see Supplementary material online, *Table S1*).

Information on the covariates included in the logistic regression models were complete, and since only a small proportion (3.8% of the total 5594) of participants had missing information on the potential mediating factors (bystander CPR and initial heart rhythm), we performed a complete-case analysis to estimate the associations.^32^ The statistical significance level was set at 0.05 *P*-value. Statistical analyses were conducted using Stata 17 software (StataCorp, College Station, TX).

## Results

### Characteristics of study population

**Figure 1:**
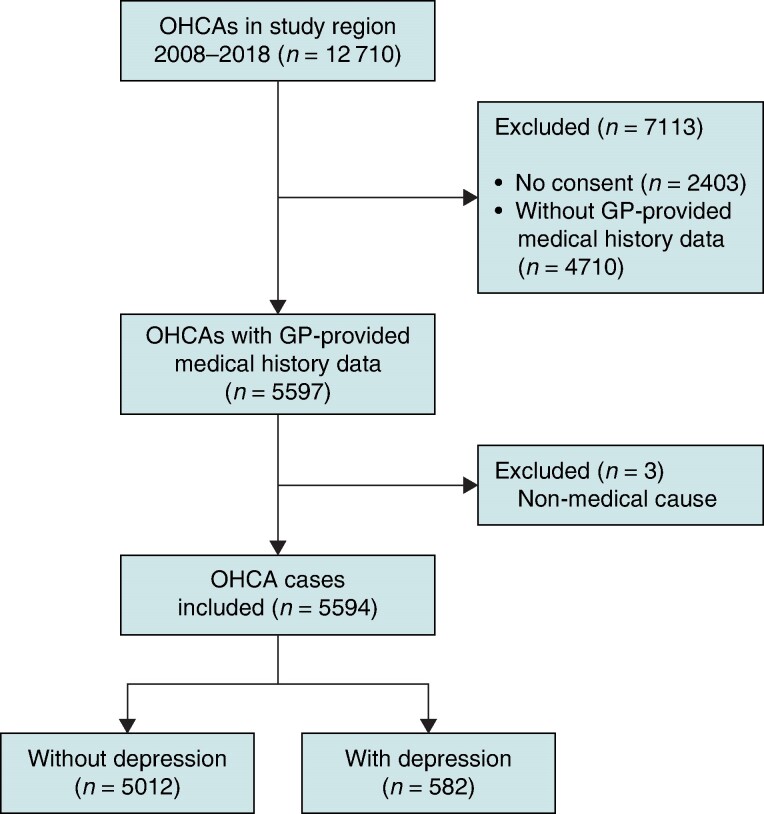
Flow diagram of the study population of people with an out-of-hospital cardiac arrest (OHCA) in the North Holland province of The Netherlands, 2008–2018. GP, general practitioner; OHCA, out-of-hospital cardiac arrest.

Between 2008 and 2018, a total of 12 710 adult OHCA cases were documented in the study region. Of these, 5594 patients with GP data were included in the analysis (*Figure 1*). A total of 4713 cases were excluded due to missing GP data. The baseline demographic and OHCA characteristics of these excluded cases were similar to those of the included patients (see Supplementary material online, *Table S1*). Of the included participants, 582 individuals (10.4%) had a documented medical history of depression prior to the OHCA event.

Patients with depression were slightly younger than those without depression, with a median (IQR) age at OHCA of 64 (55–78) and 69 (59–78) years, respectively (*Table 1*). Overall, the majority of OHCA patients (71.6%) were male, but the proportion of females in the depression group was higher compared with the non-depression group (40.7 vs. 27.0%, respectively). There was no difference in proportion with CVD comorbidity or total number of comorbidities between the two groups. The majority of patients in both groups had at least one comorbidity, and ∼30% in each group had a CVD comorbidity. Furthermore, patients with or without depression had the same EMS response time (delay between receipt of alarm call to arrival at OHCA location). Patients in the depression group had characteristics of the OHCA event which were less favourable (i.e. associated with lower chances of survival) than patients in the non-depression group, with higher proportions of unwitnessed arrests, non-shockable rhythms and OHCA in private settings, and lower proportions of CPR provision and ROSC (*Table 1*). The proportion of patients who survived longer than 30 days after OHCA or were discharged from hospital alive was lower among patients with depression (18.6%) compared with those without depression (25.1%).

**Table 1:** Baseline patient and event-related characteristics of people with an out-of-hospital cardiac arrest (OHCA) in the North Holland province of The Netherlands, 2008–2018, with or without depression

Characteristic	All 5594	People without depression 5012 (89.6)	People with depression 582 (10.4)
Age, median (IQR) years	69 (59–78)	69 (59–78)	64 (55–78)
Male (%)	4006 (71.6)	3661 (73.0)	345 (59.3)
Physical comorbidity (%)			
None	1741 (31.2)	1567 (31.3)	174 (31.0)
One	1310 (23.5)	1171 (23.4)	139 (24.7)
Two or more	2523 (45.3)	2274 (45.4)	249 (44.3)
CVD comorbidity	1650 (29.7)	1489 (29.8)	161 (28.7)
OHCA location			
Home	3863 (71.1)	3451 (70.9)	412 (72.8)
Public	1370 (25.2)	1253 (25.7)	117 (20.7)
Nursing home or other long-term care facility	201 (3.7)	164 (3.4)	37 (6.5)
Delay between call and arrival on scene, median (IQR) min	8.3 (6.4–11.0)	8.3 (6.3–11.0)	8.3 (7.0–11.0)
Witnessed arrest	4147 (74.8)	3742 (75.2)	405 (70.9)
Received bystander CPR	4027 (72.9)	3633 (73.3)	394 (69.2)
Initial shockable heart rhythm	2595 (47.6)	2390 (48.8)	205 (36.8)
Return of spontaneous circulation	2039 (40.9)	1854 (41.4)	185 (36.9)
Survived at 30-days or hospital discharge	1368 (24.5)	1260 (25.1)	108 (18.6)
Utstein comparator group^a^	2213 (39.6)	2047 (40.8)	166 (28.5)
Survived at 30 days or hospital discharge in Utstein comparator group	1078 (48.7)	1009 (49.3)	69 (41.6)

Numbers indicate *n* (%), unless noted otherwise.

IQR, interquartile range.

^a^Including patients with a bystander-witnessed collapse and an initial shockable rhythm.

Around 2213 (39.6%) of the study population had a witnessed arrest and initial shockable rhythm and were included in the Utstein comparator group sensitivity analysis. This analysis revealed that the proportion of patients in the Utstein comparator group was lower in the depression group (28.5%) than in the non-depression group (40.8%). Moreover, patients with depression had a lower proportion of survival compared with patients without depression among those included in the Utstein comparator group (41.6 vs. 49.3%, respectively).

### The association between depression and out-of-hospital cardiac arrest survival

**Figure 2:**
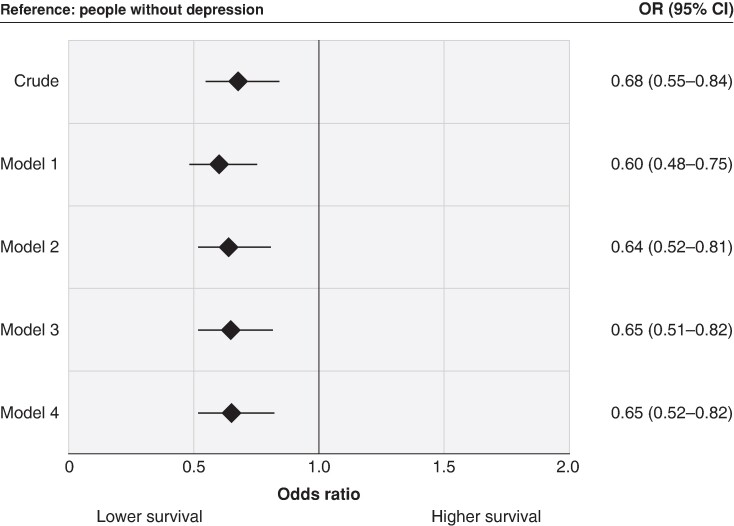
Association between depression and survival to hospital discharge or 30-day survival following out-of-hospital cardiac arrest. Odds ratios were adjusted for age (Model 1), age and sex (Model 2); age, sex, and cardiovascular disease comorbidities (Model 3); and age, sex, and number of comorbidities (Model 4). CI, confidence interval; OR, odds ratio.

**Figure 3:**
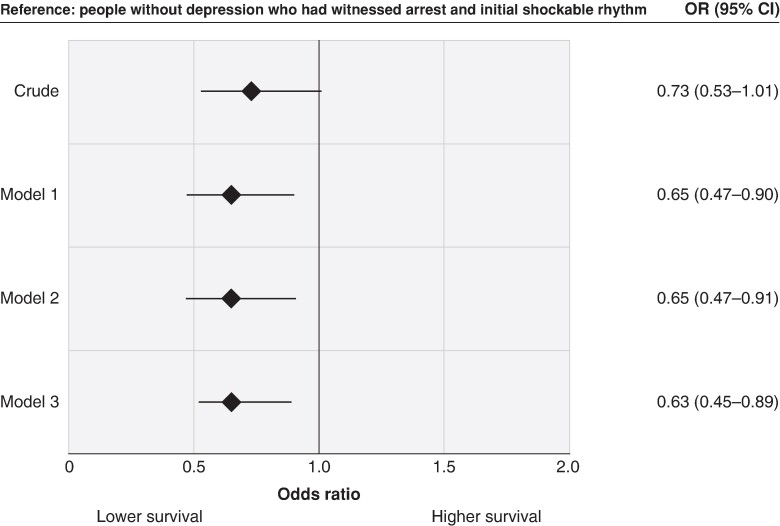
Association between depression status and out-of-hospital cardiac arrest survival of patients included in the Utstein comparator group. Odds ratios were adjusted for age (Model 1); age and sex (Model 2); and age, sex, and number of comorbidities (Model 3). CI, confidence interval; OR, odds ratio. The Utstein comparator group included 2047 patients without depression and 166 patients with depression.

**Figure 4:**
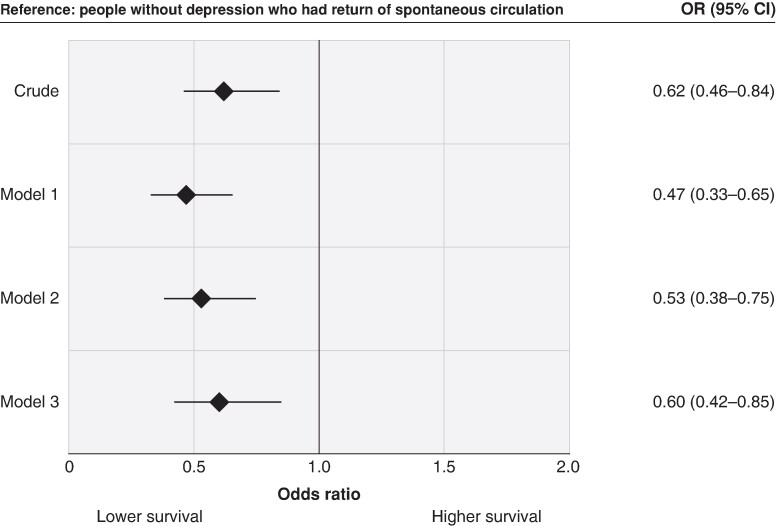
Association between depression status and out-of-hospital cardiac arrest survival of patients who had return of spontaneous circulation. Odd ratios were adjusted for age (Model 1); age and sex (Model 2); and age, sex, and number of comorbidities (Model 3). CI, confidence interval; OR, odds ratio. Included 1854 individuals without depression and 185 individuals with depression.

Compared with individuals without depression, those with depression had lower odds of survival before adjustment for any other characteristics (OR 0.68, 95% CI 0.55–0.84). The estimate declined only slightly after adjusting for age and sex (Model 2; adjusted OR 0.64, 95% CI 0.52–0.81) and did not materially change after additional adjustment for number of comorbidities (Model 3) or CVD comorbidity (Model 4; *Figure 2*). Moreover, when the analysis was restricted to the Utstein comparator group, there was a similar association between depression and reduced odds of survival after controlling for age, sex, and number of comorbidities (OR 0.63, 95% CI 0.45–0.89; *Figure 3*). We also found a similar association in our sensitivity analyses where we restricted our analyses to patients who achieved ROSC (adjusted OR 0.60, 95% CI 0.42–0.85; *Figure 4*). Furthermore, the association was significant in the additional exploratory models that adjusted for characteristics which may lie on the causal pathway, such as witnessed arrest, bystander CPR, initial rhythm, and location of arrest (Models 5–7; Supplementary material online, *Figure S2*).

### Depression and survival after out-of-hospital cardiac arrest: effect modification by sex and age

When comparing the odds of survival by sex, both males and females with depression had lower odds after adjusting for age, with similar magnitude of the association. Furthermore, there was no statistically significant evidence of effect modification between depression and survival by sex on either the additive scale (*P* = 0.156) or the multiplicative scale (*P* = 0.865) (*Table 2*).

**Table 2:** Modification of the effect of depression on survival rates after out-of-hospital cardiac arrest in the North Holland province of The Netherlands, 2008–2018, stratified by sex

Sex	Without depression (5012)		With depression (582)		OR for depression within strata of sex
	*n* survived/died	OR (95% CI)	*n* survived/died	OR (95% CI)	
Male (*n* = 4006)	1032/2629	Reference 1.0	77/268	0.63 (0.49–0.84)	0.65 (0.49–0.85)
Female (*n* = 1588)	228/1123	0.56 (0.47–0.66)	31/206	0.37 (0.25–0.55)	0.65 (0.43–0.98)

Relative excess risk due to interaction (RERI): 0.17 (−0.07 to 0.42); *P* = 0.1559. Multiplicative: OR 1.04 (0.64–1.70); *P* = 0.865. Adjusted for age.

CI, confidence interval; OR, odds ratio.

When stratifying by age group, individuals aged 65 or younger had an OR of 0.64 (95% CI 0.49–0.84), while individuals above 65 had an OR of 0.55 (95% CI 0.36–0.84). However, the 95% CIs in both age groups overlapped, and there was no statistically significant indication of effect modification between depression and survival by age on the additive scale (*P* = 0.105) or the multiplicative scale (*P* = 0.396; *Table 3*).

**Table 3:** Modification of the effect of depression on survival rates after out-of-hospital cardiac arrest in the North Holland province of The Netherlands, 2008–2018, stratified by age group

Age	Without depression (5012)		With depression (582)		OR for depression within strata of age
	*n* survived/died	OR (95% CI)	*n* survived/died	OR (95% CI)	
≤65 (*n* = 2289)	733/1245	Reference 1.0	83/228	0.66 (0.50–0.86)	0.64 (0.49–0.84)
>65 (*n* = 3305)	527/2507	0.37 (0.32–0.42)	25/246	0.20 (0.13–0.30)	0.55 (0.36–0.84)

Relative excess risk due to interaction RERI: 0.17 (−0.03 to 0.37); *P* = 0.1050. Multiplicative: OR 0.80 (0.49–1.33); *P* = 0.396. Adjusted for sex.

CI, confidence interval; OR, odds ratio.

### Bystander cardiopulmonary resuscitation and initial rhythm

Our mediation analysis suggested that 27.4% of the association between depression and lower survival rate is mediated by the fact that patients with depression more often presented with a non-shockable rhythm, and 7% is mediated by the less frequent bystander CPR provision in patients with depression.

## Discussion

### Summary of findings

We found that OHCA patients with depression presented more frequently with unfavourable OHCA characteristics but a comparable proportion of comorbidities (including CVD) and had lower 30-day survival or survival to hospital discharge than OHCA patients without depression. The association between depression and lower survival rate was similar for males and females and slightly more pronounced in older compared with younger age groups. This association persisted after controlling for the key OHCA and patient characteristics that influence survival, suggesting that depression may influence OHCA survival independently of these factors.

### Comparison with findings from other studies

Although previous studies have found an association between depression and poorer survival chances from other acute cardiac events such as myocardial infarction,^33,34^ there is limited literature on the relationship between depression and OHCA outcomes.^9,10^ Our findings on the disparities in OHCA characteristics and survival rate between individuals with vs. without depression represent a novel contribution to the existing literature. To our knowledge, only one previous study examined OHCA outcomes in individuals with psychiatric disorders, including depression.^10^ The previous study in the Danish population might be comparable to our study, but did not specifically study patients with depression. Some of our findings on the OHCA characteristics are consistent with those of the Danish study, such as patients with depression being more likely to have a non-shockable rhythm, an OHCA at home, and unwitnessed arrest. There were also some differences between the two studies in other characteristics such as age, sex, and comorbidity prevalence. However, despite these differences, both studies found an association between depression and lower odds of OHCA survival. The Danish study reported only adjusted models and found slightly stronger associations between depression and lower OHCA survival when adjusted for age, sex, and year of arrest (OR 0.41, 95% CI 0.32–0.53), compared with our study (OR 0.64, 95% CI 0.52–0.81). Out-of-hospital cardiac arrest patients with depression in the Danish study were older, with a median age of 75 (interquartile range 64–83), more likely to be female (51.7%), and had a higher prevalence of comorbidities than OHCA cases with no psychiatric disorders. The stronger association found in the Danish study may be partially attributed to the older age and higher burden of comorbidities among its cohort of patients with depression, consequently leading to poorer outcomes.^10,35^

Intriguingly, individuals with depression in the Danish cohort had a higher rate of receiving bystander CPR compared with those without psychiatric disorders, although this did not result in improved survival in this group. A few other studies have examined the association between other psychiatric conditions and long-term stress, reporting an increased incidence of OHCA in these groups and also lower OHCA survival.^36–38^

### Potential mechanisms and implications

We hypothesize that the mechanisms that may underlie the association between depression and poor OHCA survival are multifactorial and non-mutually exclusive. One key potential mechanism is the association of depression with less favourable OHCA factors, notably the lower proportions of receipt of bystander CPR and of shockable heart rhythms, which are known determinants of poor OHCA survival.^1,3^ Receipt of bystander CPR is associated with a two- to three-fold increase in 30-day survival rates following OHCA in the general population.^3^ In the general population, bystander CPR provision is influenced by several factors, such as the location of the arrest, witness status, and socioeconomic factors.^22,39–41^ Individuals with depression have less advantage in these specific aspects, such as higher proportions of unwitnessed arrests and OHCAs in private locations, both reducing their chances of receiving CPR from bystanders. Another potential mechanism is the effect of antidepressant drug use. This mechanism may be particularly relevant as the prevalence of antidepressant prescriptions nearly doubled between 1996 and 2012 in the Netherlands, from 35.5/1000 patients in 1996–1997 to 69.8/1000 patients in 2012.^42^ A previous study found that individuals who redeemed a prescription of antidepressants have an increased risk of OHCA.^43^ Another study found an association between depression and pulseless electrical activity, a type of non-shockable rhythm.^44^ In particular, the use of tricyclic antidepressants has been associated with the blockage of cardiac sodium channels, which are key drivers of cardiac excitability.^45,46^ Furthermore, the complex medication profiles of individuals with depression may affect cardiac excitability as multiple (psychoactive) drugs block cardiac depolarization.^47^ This may promote the more rapid dissolution from shockable to non-shockable rhythms and ultimately contribute to lower survival chances in this population.^12,48,49^ Moreover, it is theoretically possible that depolarization-blocking drugs reduce the amplitude spectrum area (AMSA) of the ventricular fibrillation signal. If that is the case and defibrillation protocols do not take AMSA into account, patients with low AMSA may inadvertently receive inappropriate defibrillation attempts instead of continued chest compression; this may result in reduced survival chances.^50^ Unfortunately, at the time of this study, we were unable to obtain full prescription data of the study cohort that would allow us to test these possible mechanisms. In any case, factors in addition to the higher proportion of non-shockable rhythm among patients with depression may play a role as, in our study, OHCA survival rate remained significantly lower among patients with depression in the Utstein comparator group (who had shockable initial rhythm by definition), suggesting an additional risk unexplained by the initial heart rhythm (or witness status).

Depression is also linked to a range of behavioural and lifestyle factors that can affect physical health and increase the risk of poor outcomes from physical conditions, such as unhealthy diet, smoking, lack of exercise, and substance abuse.^51^ These factors may partially account for the overall increased risk of physical diseases and premature mortality observed in individuals with depression compared with the general population.^20^ However, in our study, we did not observe differences in the number of comorbidities between people with or without depression. Nevertheless, it is noteworthy that many of these behavioural and lifestyle factors, such as smoking and lower adherence to treatment, influence not only the risk of developing physical diseases but also the unfavourable outcomes associated with these conditions.^51–54^ Consequently, among people with depression who have physical comorbidities, these factors might reduce the efficacy of OHCA treatment, leading to poorer prognosis.^21,52–54^

The poorer OHCA survival in patients with depression was observed even among those who achieved ROSC, suggesting an increased risk of in-hospital mortality for OHCA patients with depression. This may be linked to higher rates of complications during post-OHCA management in hospitals or disparities in healthcare provision post-OHCA. Several studies have demonstrated disparities in healthcare provision for individuals with depression, even in cases of life-threatening conditions.^33,55^ For instance, in Scotland, severe depression has been associated with significantly lower rates of coronary revascularization following a myocardial infarction.^33^

Understanding the pathophysiological pathways of association is crucial for developing tailored pre-hospital and in-hospital clinical care models for OHCA patients with depression. Furthermore, targeting communities with a higher prevalence of depression and lower CPR rates, such as communities with lower socioeconomic status, for arrest recognition and CPR education campaigns could increase survival rates in these populations.^22,56^

Due to the abrupt and unpredictable nature of OHCA, randomized clinical trials are less feasible in the context of OHCA. Thus, it is necessary to combine individual-level data from different sources to conduct more robust observational studies in future research. Future studies should attempt to identify mechanisms that affect OHCA survival in patients with depression, including lifestyle factors, physiological mechanisms, and other potential factors such as the use of antidepressants. Further research is also warranted to identify other possible risk factors for OHCA incidence in the whole population and the people at risk of poor outcomes, like people with other psychiatric disorders.

### Strengths and limitations

A strength of our study is the population-based design, which included a large and unselected number of OHCA patients from an OHCA registry with relevant OHCA information, reducing the risk of selection bias. Nevertheless, we excluded OHCA patients whose GPs did not respond to the medical history questionnaires, which in turn restricted the number of participants included in our analysis. Among our study population, individuals whose GPs did not respond had a lower OHCA survival rate in comparison with the rest of the cohort. However, there was no difference in baseline demographic and OHCA characteristics between these excluded cases and those who were included. Consequently, it is possible that our study included relatively more patients with favourable outcomes, but it is unknown whether the included patients have a different depression prevalence from the excluded participants. However, a potential difference in depression prevalence between included and excluded people is unlikely to invalidate the observed association between depression and OHCA survival in our study since we excluded all patients whose GPs did not respond from our analysis non-differentially. One limitation is the lack of specific information on how GPs diagnose depression (this information was not included in the questionnaires that the ARREST team requested from the GPs). Furthermore, while the list of comorbidities included in the questionnaires is extensive, it does not cover all comorbidities, potentially leaving some residual confounders unaccounted for. Another limitation of our study is the lack of information on socioeconomic status, which is associated with an increased risk of both depression and OHCA incidence and poor OHCA outcomes.^7,22,41,56,57^ A previous study conducted in the Netherlands identified an association between low socioeconomic status and lower OHCA survival rates^41^ but suggested that this association was not explained by the differences in OHCA characteristics or comorbidities by socioeconomic status. Our study’s findings regarding the association between depression and OHCA survival could offer a potential pathway for the observed link between socioeconomic status and reduced OHCA survival. However, we plan to examine the interplay between these factors in future studies.

## Conclusion

We found that OHCA patients with depression presented more frequently with unfavourable OHCA characteristics, but with a similar number of comorbidities, including CVD, compared with OHCA patients without depression. Patients with depression had reduced chances of OHCA 30-day survival or survival to hospital discharge compared with those without depression. This association persisted after controlling for key OHCA and patient characteristics that influence OHCA survival, suggesting that the link between depression and lower OHCA survival is independent of these key factors.

## Supplementary Material

euae209_Supplementary_Data

## Data Availability

The data cannot be shared publicly for privacy of individuals who participated in the study as data cannot be provided completely anonymous according to the Medical Ethics Committee and the Data Protection Officer of our institution. A formal application must be made to access the dataset (MEC: mecamc@amc.nl, DPO: fg@amc.nl).
