# Supplementary material for: The association of depression and patient and resuscitation characteristics with survival after out-of-hospital cardiac arrest: a cohort study
Source: Europace. 2024 Aug 6;26(8):euae209. doi: 10.1093/europace/euae209 (PMC11337125; doi:10.1093/europace/euae209)
Supplement: euae209_Supplementary_Data [file euae209_supplementary_data.docx]

**Table S1 Characteristics of people with an out-of-hospital cardiac arrest (OHCA) in the North-Holland province of the Netherlands, 2008-2018 with or without general practitioner (GP) records**

|  | All  10,307 | Available GP data  5,594 (54.3) | Missing GP data  4,713 (45.7) |
| --- | --- | --- | --- |
| Age, median (IQR) years | 67 (57-77) | 69 (59-78) | 66 (55-77) |
| Sex | | | |
| Female | 3,091 | 1,588 (28.4) | 1,503 (31.9) |
| Male | 7,211 | 4,006 (71.6) | 3,205 (68.1) |
| OHCA Location | | | |
| Home | 7,215 | 3,863 (69.1) | 3,352 (72.5) |
| Public | 2,393 | 1,370 (24.5) | 1,023 (22.1) |
| Nursing home or other long term care facility | 452 | 201 (3.6) | 251 (5.4) |
| Arrest witness status | | | |
| Non witnessed | 2,820 | 1,398 (25.0) | 1,422 (30.4) |
| Witnessed | 7,397 | 4,147 (74.1) | 3,250 (69.6) |
| 30-day survival or survival to hospital discharge |  |  |  |
| Yes | 2,003 (19.4) | 1,368 (24.5) | 635 (13.5) |
| No | 8,304 (80.6) | 4,226 (75.5) | 4,078 (86.5) |

Numbers indicate N (%), unless noted otherwise. The sum may not add up to the total because of missing values.

IQR: interquartile range


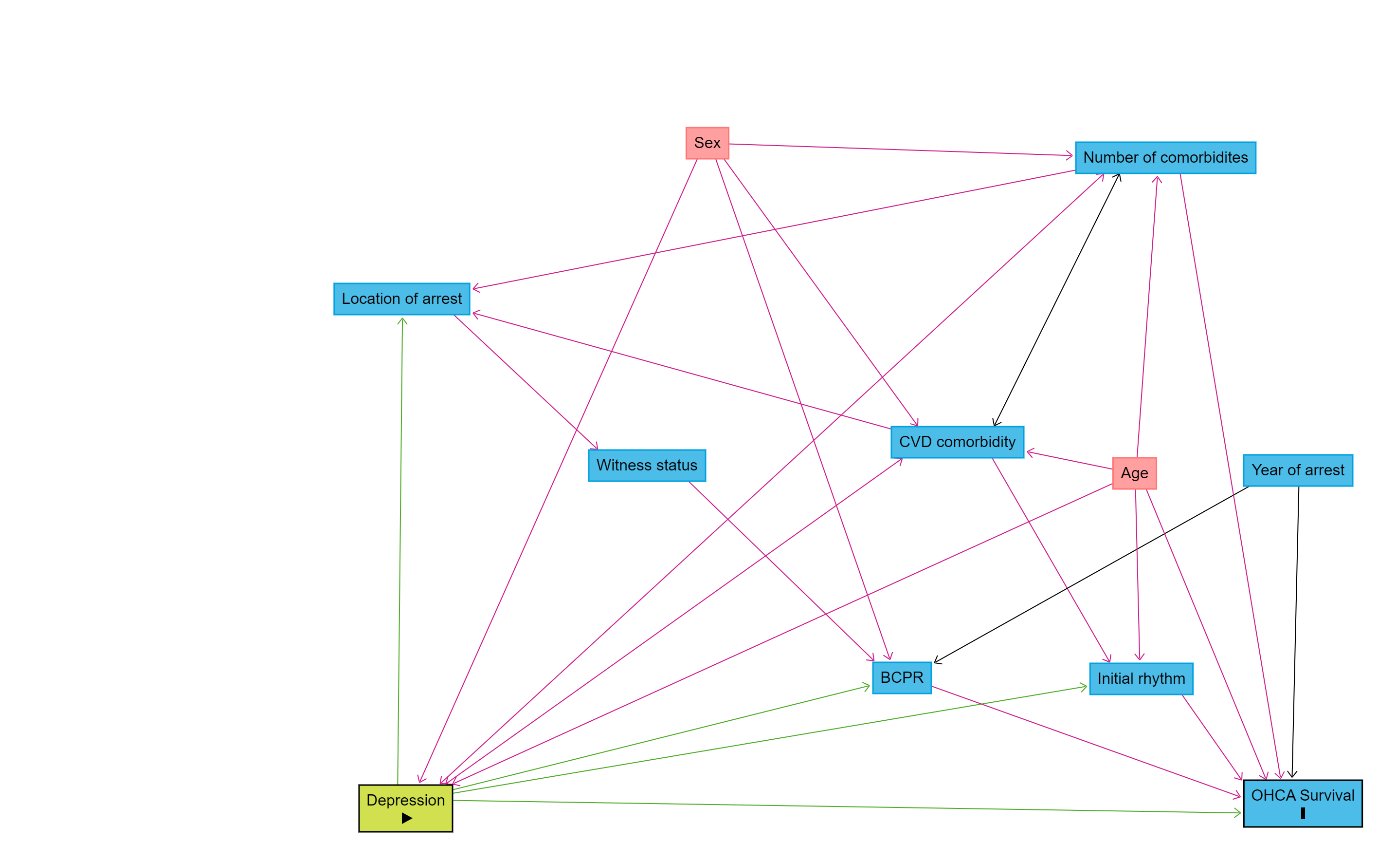
**Figure A1: Directed acyclic graph of the assumed association between depression out-of-hospital cardiac arrest (OHCA) 30-day survival or survival to hospital discharge**

BCPR: bystander cardiopulmonary resuscitation; CVD: cardiovascular disease; OHCA: out-of-hospital cardiac arrest


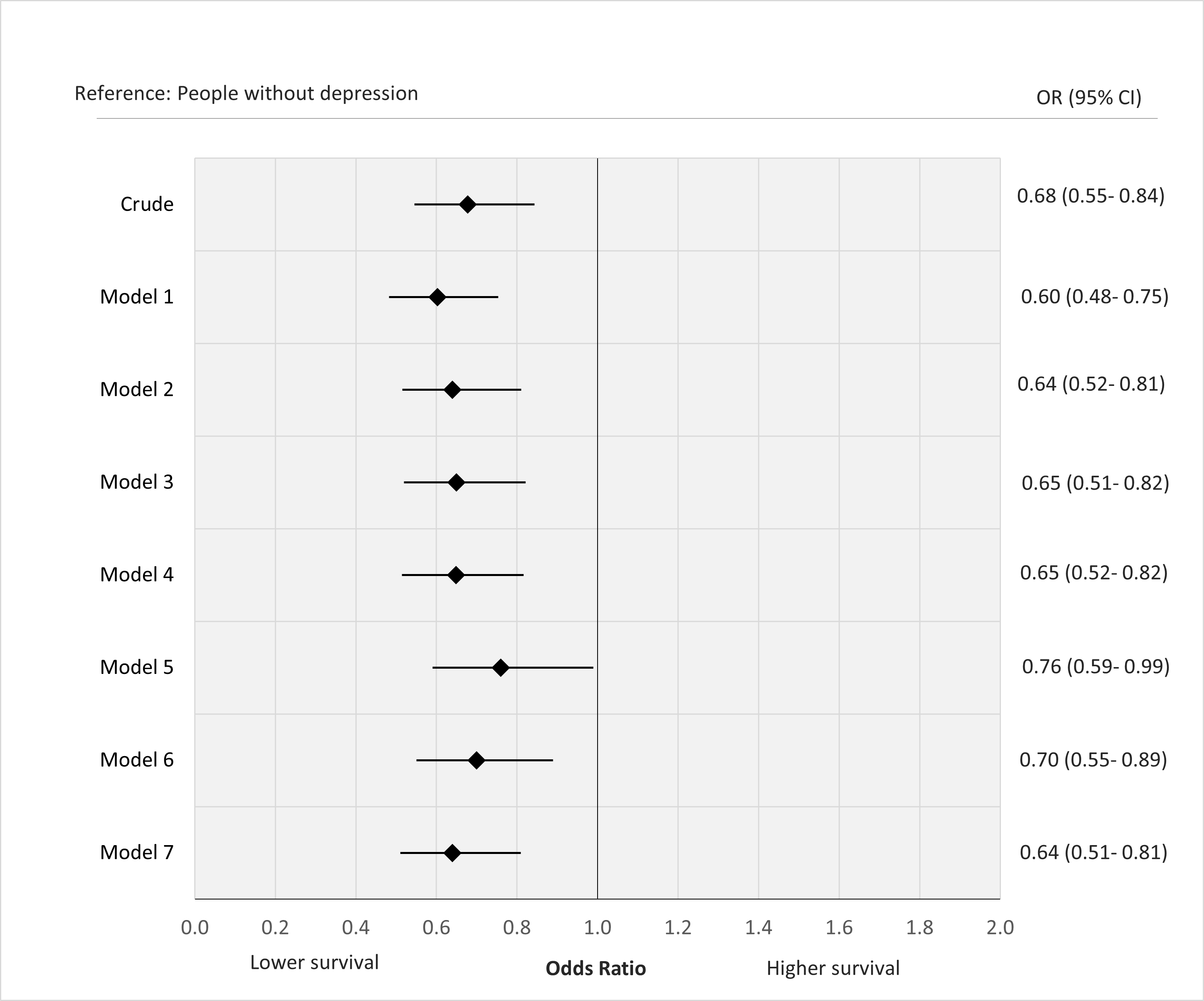


**Figure A2 Additional models exploring the association between depression and the survival to hospital discharge or the 30-day survival after an out-of-hospital cardiac arrest were examined. ORs were adjusted for: age (Model 1), age and sex (Model 2), age, sex and cardiovascular disease comorbidities (Model 3), age, sex and number of comorbidities (Model 4), age, sex, number of comorbidities and cardiovascular disease comorbidities (Model 5), age, sex, bystander CPR and initial rhythm (Model 6), age, sex, location of arrest and witness status (Model 7).**

CI: Confidence Interval; OR: Odds Ratio
